# Circulating Tryptase as a Marker for Subclinical Atherosclerosis in Obese Subjects

**DOI:** 10.1371/journal.pone.0097014

**Published:** 2014-05-15

**Authors:** María Moreno, Josep Puig, Marta Serrano, José María Moreno-Navarrete, Francisco Ortega, Wifredo Ricart, Jose Manuel Fernandez-Real

**Affiliations:** 1 Department of Diabetes, Endocrinology and Nutrition, Institut d'Investigació Biomèdica de Girona (IdIBGi), CIBEROBN (CB06/03/010) and Instituto de Salud Carlos III (ISCIII), Girona, Spain; 2 Department of Radiology, Institut d'Investigació Biomèdica de Girona (IdIBGi), CIBEROBN (CB06/03/010) and Instituto de Salud Carlos III (ISCIII), Girona, Spain; King's College London School of Medicine, United Kingdom

## Abstract

**Introduction:**

Mast cells participate in atherogenesis by releasing cytokines to induce vascular cell protease expression. Tryptase is expressed highly in human atherosclerotic lesions and the inhibition of tryptase activity hampers its capacity to maintain cholesterol inside macrophague foam cells. We aimed to investigate the association between circulating tryptase levels and subclinical atherosclerosis through estimation of carotid intima-media thickness (c-IMT) as surrogate marker for increased cardiovascular risk in obese and non-obese subjects.

**Methods:**

Circulating tryptase levels (ELISA) and metabolic parameters were analyzed in 228 subjects. Atherosclerosis (c-IMT>0.9 mm) was evaluated ultrasonographically.

**Results:**

Significant positive associations were evident between circulating tryptase levels and BMI, fat mass, glycated haemoglobin, fasting insulin, HOMAIR, fasting triglycerides and ultrasensitive PCR (p<0.05 from linear-trend ANOVA). The positive association between tryptase levels and insulin resistance parameters, suggested a glucose homeostasis impairment in individuals with higher tryptase levels. The negative asociation between tryptase levels and HDL-cholesterol supports the proatherogenic role of this protease (p<0.0001). Circulating tryptase levels were strongly associated with c-IMT measurements (p<0.0001 from linear-trend ANOVA), and were higher in subjects with presence of carotid plaque (p<0.0001). Tryptase levels (beta = 0.015, p = 0.001) contributed independently to subclinical atherosclerosis variance after controlling for cardiovascular risk factors (BMI, blood pressure, LDL-cholesterol).

**Conclusions:**

Circulating tryptase level is associated to obesity related parameters and has a close relation with various metabolic risk factors. Moreover, serum tryptase level was independently associated with c-IMT, suggesting its potential use as a surrogate marker for subclinical atherosclerosis in obese subjects.

## Introduction

Atherosclerosis is a chronic inflammatory disease that is the main cause of cardiovascular morbidity and mortality all over the world. It is characterized by the progressive accumulation of cholesterol in the intimal layer of arterial walls of large- and medium-sized arteries, leading to the formation of plaques and vascular obstruction [1], [2]. Inflammatory cells such as lymphocytes, macrophages, neutrophils, and mast cells are involved in the pathogenesis of atherosclerotic plaque rupture, as they cause the fibrous plaque to weaken because of the enzyme activity of the leukocytes that degrade the extracellular matrix [3]. Mast cells are derived from pluripotent hematopoietic stem cells, which are released in the blood flow, and then migrate to the tissue where they proliferate, differentiate, and become resident [4], [5]. Two types of mast cells, differing in neutral (cytoplasmic) proteases, are identified: mast cells that contain tryptase and mast cells that contain tryptase and chymase [6]. Importantly, these proteases were found in the human arterial intima twenty years ago, both normal and atherosclerotic [7].

Tryptase is a trypsin-like serine proteinase which has been estimated to constitute approximately 20% of the total cellular protein of human mast cells [8]. This is stored fully active in the cytoplasmic granules of all human mast cells and is released in the peripheral circulation [9]. The physiologic role of tryptase is still uncertain, however the activity of the enzyme is only observed in damaged tissues such as those of people with atherosclerosis [10]. Also, mast cells are locally activated and release tryptase into their microenvironment, where active tryptase can act on the various extracellular targets, i.e. activate pro-MMPs, and degrade lipoproteins and fibronectin [11]. Mast cell activation in atherosclerosis has been demonstrated to promote intraplaque haemorrhage resulting in plaque progression and destabilisation. In this sense, several studies have established an association of blood tryptase levels with atherosclerotic plaque instability [12], [13]. A recent paper performed in ApoE-/- mice, describes the role of tryptase in atherosclerotic progression and intraplaque hemorrhage [14]. Indeed, tryptase activates pro-metalloproteinases and chemokines, and degrades lipoproteins and fibronectin [15], [16]. In a study of aortas sections obtained from autopsies has been shown that the degree of macroscopic lesion of atherosclerosis increased proportionally with the increase in the density of mast cell chymase and tryptase [17]. In this sense, in a recent paper mast cells are associated with plaque microvessel density [18]. Evidence derived from human data supports an association of mast cells and obesity since obese subjects had higher serum tryptase levels and an increased number of mast cells stained with tryptase than lean individuals in white adipose tissue [19]–[21]. In the same setting, tryptase has also been associated with older age, fasting glucose, total- and LDL-cholesterol and fasting triglycerides [12].

In recent years, there has been growing interest in identifying asymptomatic individuals with increased cardiovascular risk who may benefit from specific primary prevention. It is well-known that increased carotid intima-media thickness (c-IMT), measured by B-mode ultrasonography, constitutes an independent risk marker for coronary artery disease and stroke, becoming a sensitive subclinical atherosclerosis marker [22]. The purpose of our study was to investigate the association between circulating tryptase levels and subclinical atherosclerosis through estimation of c-IMT as a surrogate marker for increased cardiovascular risk.

## Materials and Methods

### Subjects

From January 2010 to February 2012, we consecutively recruited 228 subjects from the ongoing multicenter FLORINASH Project, undertaken to evaluate the role of intestinal microflora in adults with NAFLD (non-alcoholic fatty liver disease). Inclusion criteria were age 30 to 65 years, and ability to understand study procedures. Exclusion criteria were systemic disease, infection in the previous month, serious chronic illness, >20 g ethanol intake per day, or use of medications that might interfere with insulin action. 19 subjects were taking statins. No significant differences in tryptase levels were seen according to statin treatment. 72.6% of the population was non-smoker. Circulating tryptase levels were higher among smokers (13.67±6.7 *vs* 11.51±5.9 p = 0.022). All subjects gave written informed consent, validated and approved by the ethical committee of the Hospital Universitari Dr. Josep Trueta (Comitè d'Ètica d'Investigació Clínica, CEIC), after the purpose of the study was explained to them. Ethical committee of the Hospital Universitari Dr. Josep Trueta specifically approved this study (ethical approval number 2009046).

### Analytical methods

Each patient underwent anthropometric measurements, vascular ultrasound and laboratory parameters on the same day. After 8 h fasting, blood was obtained for measurement of plasma lipids, glucose, and insulin. Serum glucose concentrations were measured in duplicate by the glucose oxidase method using a Beckman glucose analyser II (Beckman Instruments, Brea, California). Intraassay and interassay coefficients of variation were less than 4% for all these tests. We used a Roche Hitachi Cobas c 711 instrument to do the determinations. Total serum cholesterol was measured by an enzymatic, colorimetric method through the cholesterol esterase / cholesterol oxidase / peroxidase reaction (Cobas CHOL2). HDL cholesterol was quantified by an homogeneous enzymatic colorimetric assay through the cholesterol esterase / cholesterol oxidase / peroxidase reaction (Cobas HDLC3). Total serum triglycerides were measured by an enzymatic, colorimetric method with glycerol phosphate oxidase and peroxidase (Cobas TRIGL). LDL cholesterol was calculated using the Friedewald formula. Glycated haemoglobin (HbA1c) was measured by high-pressure liquid chromatography with the use of a fully automated glycosylated hemoglobin analyzer system (Hitachi L-9100). C-reactive protein (ultrasensitive assay; Beckman, Fullerton, CA) was determined by a routine laboratory test, with intra- and interassay coefficients of variation <4%. The lower limit of detection is 0.02 mg/l.

Serum insulin was measured in duplicate in the same centralized laboratory by a monoclonal immunoradiometric assay (Medgenix Diagnostics, Fleunes, Belgium). The intra-assay CV was 5.2% at a concentration of 10 mU/l and 3.4% at 130 mU/l. The inter-assay CVs were 6.9 and 4.5% at 14 and 89 mU/l, respectively. Insulin resistance was determined by the homeostasis model assessment of insulin resistance (HOMA_IR_).

Both types of tryptase (alpha and beta forms) levels were measured in serum by a commercial ELISA Kit (Human tryptase alpha/beta-1, Tryptase-1 Elisa Kit. Catalog No: E1070h; EIAab, China. http://www.eiaab.com/entries/steps/ELISA%20Kit/TRYB1_HUMAN/Human). The intra-assay coefficient of variation was ≤4.1% and the inter-assay coefficient of variation was ≤7.9%. Tryptase activity has not been tested.

### Body composition

Fat mass was determinated by dual energy x-ray absorptiometry (DEXA), using a Lunar Prodigy Full Oracle (GE Healthcare, enCore software version 13.2). Whole body composition (fat mass, fat-free soft tissue mass) was obtanined according to standard procedures, by trained personnel. Body fat composition was also estimated in those subjects by Bio-electrical impedance analysis (BC-418, Tanita Corporation of America, Illinois, USA). Obesity was defined as BMI >30 kg/m^2^.

### Ultrasound evaluation

We used a Siemens Acuson S2000 (Mochida Siemens Medical System, Tokyo, Japan) ultrasound system with a 3.5 MHz convex transducer to scan the liver and a 7.5 mHz linear array transducer to scan carotid arteries. Images were transferred to Starviewer software, developed in our laboratory (http://gilab.udg.edu), and independently evaluated by two radiologists blinded to clinical and laboratory data. Carotid arteries were evaluated according to the Mannheim Consensus [23]. c-IMT values were manually measured in the far wall of each common carotid artery in two locations a) in a proximal segment and b) in a plaque-free segment 10 mm from the bifurcation. Measurements were performed by two different observers. Pearson's correlation for c-IMT was 0.75. The mean c-IMT value for each subject was calculated from these four measurements. Values >0.90 mm were considered pathologically increased. Plaque was defined as a focal structure of the inner vessel wall of at least 0.5 mm or 50% of the surrounding IMT value, as well as demonstrates a thickness >1.5 mm as measured from the media-adventitia interface to the intima lumen interface any IMT measurement >1.5 mm. [23].

### Statistical analysis

Statistical analyses were performed using SPSS 12.0 software for Windows (SPSS, Chicago, IL, USA). Pearson correlation was used to determine agreement on c-IMT. Results are expressed as means ± standard deviation for continuous variables. Parameters that did not fullfil normal distribution were mathematically transformed to improve symmetry for subsequent analyses. One-way ANOVA with Bonferroni correction as the post-hoc test, were used to seek differences in clinical variables among groups. We used Student's t-test to determine differences in quantitative variables. The relation between variables was tested using Pearson's test and stepwise multiple linear regression analysis. The general linear model was also used to identify independent predictors of atherosclerosis after adjusting for cardiovascular risk factors (BMI, blood pressure or LDL-cholesterol). Receiver operating characteristic (ROC) curve analysis was used to determine the diagnostic potential. Statistical significance was set at p<0.05.

## Results

### Characteristics of the study participants

To study whether circulating tryptase levels are associated with metabolic parameters in humans, the study subjects were stratified according to tryptase quartiles. Significant positive associations were evident between circulating tryptase levels and BMI, fat mass, glycated haemoglobin, fasting insulin, HOMAIR, fasting triglycerides and ultrasensitive PCR. On the contrary, negative associations with HDL-cholesterol were observed (p<0.05 for linear-trend ANOVA for comparisons across tryptase quartiles; Table 1).

**Table 1:** Anthropometrical and clinical varibles of the study subjects according to quartiles of circulating tryptase levels (n = 228).

	Tryptase (µg/L)				
	<7.9	7.9–12.1	12.1–15.7	>15.7	*p*
** Patients/Men (%)**	57/16 (28.1)	57/24 (42.1)	57/26 (45.6)	57/27 (47.4)	0.035
** Age (years)**	43.7±11.0	43.4±10.1	43.3±10.4	45.4±8.9	ns
** BMI (kg/m^2^)**	34.3±10.6	38.3±10.9	42.6±8.5*	44.0±6.5*	<0.0001
BMI (>30 kg/m^2^) (%)	61.1	74.5	90.7	96.4	<0.0001
** Waist-to-hip ratio**					
Female	0.78±0.3	0.73±0.4	0.88±0.1	0.71±0.6	ns
Male	0.95±0.1	0.89±0.4	0.91±0.4	0.94±0.4	ns
** Fat mass (densitometry)**	38445±18334	43712±19170	51166±14554*	54486±12142*	<0.0001
** SBP (mmHg)**	130.9±18.9	136.5±17.5	137.4±22.3	137.0±16.9	ns
** DBP (mmHg)**	73.4±12.3	76.7±11.1	76.5±10.8	77.3±12.4	ns
** Fasting glucose (mmol/L)**	5.14±0.9	5.02±0.6	5.17±0.9	5.08±0.6	ns
** Glycated hemoglobin (%)**	5.5±0.3	5.6±0.4	5.7±0.4	5.6±0.4	0.043
** Fasting insulin (pmol/L)**	52.8±46.8	69.6±51.6	90.6±66.0*	93.0±57.6*	<0.0001
** HOMA_IR_**	2.0±1.8	2.7±2.1	3.8±3.3*	3.5±2.3*	<0.0001
** Total chol (mmol/L)**	5.03±0.9	4.90±0.9	5.17±1.1	5.13±0.8±30.2	ns
** HDL-chol (mmol/L)**	1.49±0.5	1.33±0.4	1.16±0.3*	1.18±0.3*	<0.0001
** LDL-chol (mmol/L)**	3.07±0.8	3.03±0.8	3.28±0.8	3.19±0.7	ns
** Triglycerides (mmol/L)**	1.04±0.5	1.17±0.5	1.48±0.7*	1.61±0.7*	<0.0001
** ultraCRP (mg/L)**	4.9±5.7	7.5±8.2	9.6±7.6*	8.2±6.3	0.006

Abbreviations: BMI, body mass index; HOMA, homeostasis model assessment.; HDL, high density lipoprotein; LDL, low density lipoprotein; ultraCRP, ultrasensitive C-reactive protein. Mean ± s.d. *p* values shown on the right are for linear trend (ANOVA). Post-hoc comparisons (Bonferroni): * *p<0.05* compared to the first quartile. ns: non-significant.

**Figure 1:**
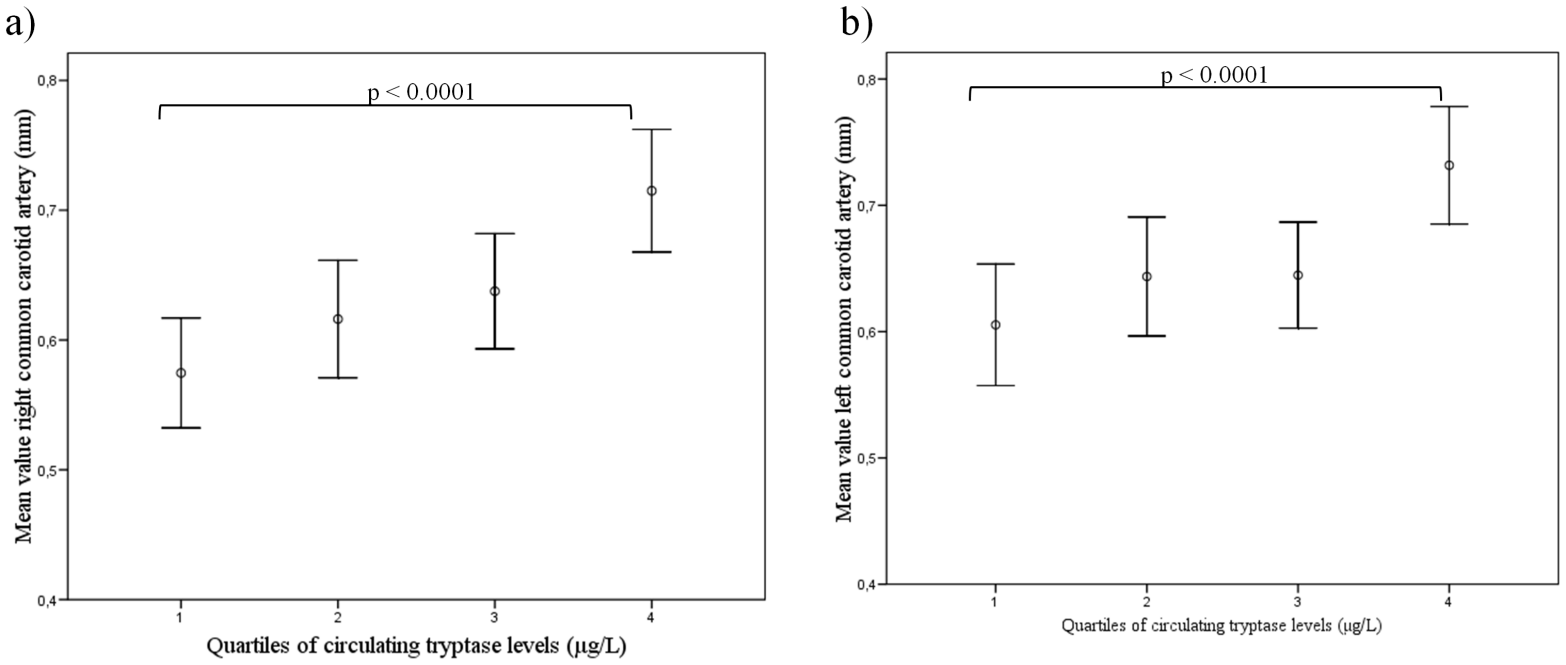
Error-bar plots of a) mean value right common carotid artery and b) mean value left common carotid artery according to quartiles of circulating tryptase levels. Plots are means and 95% CI. (p from linear-trend ANOVA) n = 228.

**Figure 2:**
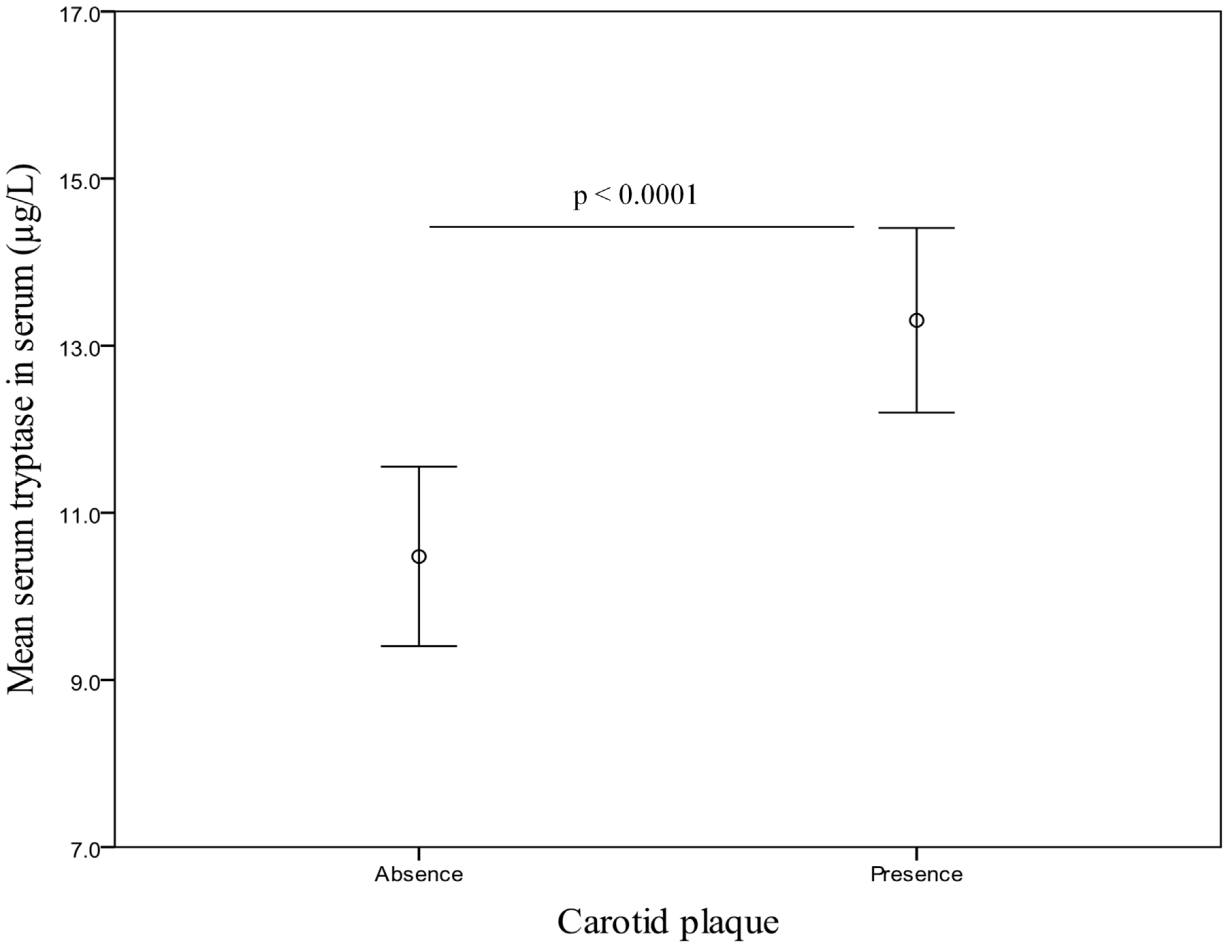
Differences in circulating tryptase levels according to carotid plaque absence (n = 100) or presence (n = 128) (p<0.0001). Plots are means and 95% CI.

**Figure 3:**
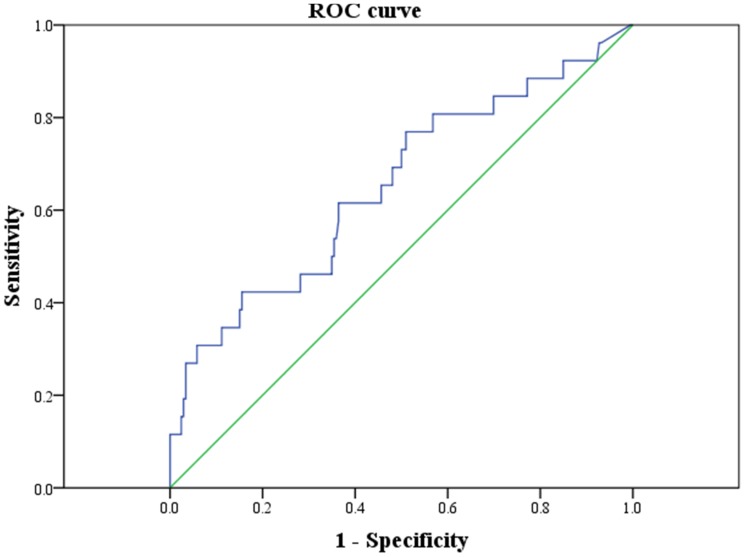
Receiver Operating Characteristic (ROC) curve for tryptase in the prediction of carotid atherosclerosis in both gender combined (AUC = 0.653 (0.532–0.774)). n = 228.

We next explored the association between circulating tryptase levels and C-IMT. As shown in figure 1, C-IMT was significantly higher in the highest quartile than in the middle or lowest circulating tryptase quartiles (p<0.05 for linear-trend ANOVA for comparisons across tryptase quartiles). Moreover, when the presence or absence of carotid plaque was evaluated, subjects with carotid plaque presence shown higher circulating tryptase level than carotid plaque absence (p<0.0001) (figure 2). Importantly, the area under the curve for circulating tryptase to predict atherosclerosis was 0.653 (0.532–0.774) in both gender combined (figure 3).

In multiple linear regression models, BMI (beta = 0.310, p = 0.001) contributed independently to circulating tryptase levels variance after controlling for age, gender and smoking. Moreover, circulating tryptase levels (beta = 0.219, p = 0.002) contributed independently to subclinical atherosclerosis variance after controlling for several cardiovascular risk factors (BMI, blood pressure, LDL-cholesterol or smoking).

## Discussion

The present study confirmed that serum tryptase level has a positive correlation with obesity and insulin resistance related parameters and with and adverse lipid profile. Most importantly, we demonstrated for the first time that circulating tryptase levels were positively associated with subclinical atherosclerosis, as represented by the c-IMT. Moreover, circulating tryptase level was an independent determining factor for the subclinical atherosclerosis, even after adjusting for other cardiovascular risk factors. Our data are in line with a previous paper reported mast cells distribution density in carotid samples was associated with an atherogenic lipid profile and high-grade of carotid artery stenosis [24].

Although tryptase is a well-known protease with an established role in immune process, recent data indicate that might provide a link between obesity and chronic inflammation. On the one hand, growing evidence derived from human data supports the link among tryptase and obesity. In the current study, there is a trend toward between circulating tryptase levels and obesity related parameters such as BMI and fat mass. These findings are in line with other authors who reported higher tryptase levels in obese subjects [18], [19]. Moreover, an increased number of mast cells stained with tryptase have been also demonstrated in scWAT of obese patients, [20] suggesting that mast cell accumulation contributes to adipose tissue inflammation and alteration of glycemic status in obese subjects.

A positive association between circulating tryptase levels and insulin resistance parameters (HbA1c, insulin and HOMA_IR_) have been also found in our study, suggesting a glucose homeostasis impairment in those individuals with higher tryptase levels. In line with our results, two Chinese studies, have considered circulating tryptase levels as an independent risk factor of pre-diabetes and diabetes mellitus [25], [26], reinforcing the close association between the immune system, obesity, and vascular diseases.

On the other hand, epidemiology, histopathology and experimental studies point toward a proatherogenic role for mast cells, involving tryptase [27]. According to the cholesterol balance theory of atherogenesis, atherosclerosis is a cholesterol storage disease of the arterial intima in which cholesterol accumulation results from an imbalance between cholesterol influx and efflux [28]. HDL_3_ particles efficiently remove cholesterol from foam cells. At this point we should highlight the pre-betaHDL-degrading effect of tryptase previously reported [29]. In our study we have found an inverse association between circulating tryptase and HDL-cholesterol levels. Tryptase mesurements have been performed in steady state conditions when the chief isoform is alpha-tryptase, responsible for degradation of the apolipoprotein A-I of prebeta HDL particles [30], [31]. However, since pre-beta form of HDL is a very small fraction of the HDL-class in the circulation and the degrading effect is likely to occur in tissues but not in the circulation, serum tryptase is unlikely to contribute significantly to low HDL-cholesterol levels observed herein. Indeed, the main contributor to the HDL-cholesterol levels variation is BMI (beta = −0.546; p = <0.0001) but not tryptase levels (beta = −0.043; p = 0.468).

In the light of our data it could be possible that adipose-derived tryptase may be involved in the pathogenesis of obesity-related inflammatory disorders, including atherosclerosis. However, the prevailing concept is that the tryptase acts in the tissue in which it is secreted but not in remote tissues into which a small fraction of the circulating tryptase may be transported across the endothelial barrier [32]. There are many questions emerged from this study, whether the adipose tissue is a critical source of tryptase levels and if it could act on atherosclerotic plaques or what the tryptase activity originating from adipose tissue is. Further studies will be needed to test the tryptase activity derived from adipose tissue.

Indeed, tryptase levels of our study are quite high compared to the reference range in healthy population. However, tryptase basal levels are associated with BMI. In agreement with our data, Liu et al. have reported similar tryptase levels in their lean vs obese population [19], suggesting that baseline levels in obese subjects are higher than the reference range in healthy population

Regarding the influence of circulating tryptase on the atherosclerotic process, few studies have addressed this question. In an autopsy cases study, the degree of atherosclerosis was positively correlated with the expression of local tryptase in the atherosclerotic plaques [16]. In this sense, the main finding of our cross-sectional study is that circulating tryptase levels are stongly associated with c-IMT measurements, being the highest tryptase levels in patients with carotid plaque. A thickened c-IMT does not immediately lead to cardiovascular events, but reflects the degree of atherosclerosis elsewhere in the arterial system [33]. Moreover our results suggests that circulating tryptase could be a useful marker as a predictor of cardiovascular disease.

In conclusion, the present study confirmed that circulating tryptase level is significantly elevated in obese individuals and is associated with various metabolic risk factors. Moreover, we demonstrated that the serum tryptase levels were independently associated with c-IMT and its potential use as a marker for subclinical atherosclerosis. Further experimental studies are warranted to clarify the role of tryptase in the atherosclerotic process.
